# Therapeutic switching: from antidermatophytic essential oils to new leishmanicidal products

**DOI:** 10.1590/0074-02760140332

**Published:** 2015-02

**Authors:** Emeline Houël, German Gonzalez, Jean-Marie Bessière, Guillaume Odonne, Véronique Eparvier, Eric Deharo, Didier Stien

**Affiliations:** 1 Centre National de la Recherche Scientifique, Unité Mixte de Recherche Ecologie des Forêts de Guyane, Institut Pasteur de la Guyane, Cayenne, French Guiana; 2 Laboratoire de Pharmacochimie et Pharmacologie pour le Développement, Unité Mixte de Recherche 152, Faculté des Sciences Pharmaceutiques, Université de Toulouse III-Paul Sabatier, Toulouse, France; 3 Laboratoire de Pharmacochimie et Pharmacologie pour le Développement, Unité Mixte de Recherche 152, Institut de Recherche pour le Développement, Toulouse, France; 4 École Nationale Supérieure de Chimie, Unité Mixte de Recherche 5076, Montpellier, France; 5 Centre National de la Recherche Scientifique-Guyane, Unité de Service et de Recherche 3456, Cayenne, French Guiana; 6 Institut de Chimie des Substances Naturelles, Centre National de la Recherche Scientifique, Gif-sur-Yvette, France; 7 Laboratoire Océanologique, Laboratoire de Biodiversité et Biotechnologies Microbiennes, Centre National de la Recherche Scientifique, Université Pierre et Marie Curie, Banyuls-sur-mer, France

**Keywords:** therapeutic switching, antifungal agents, antiparasitic agents, Leishmania, peritoneal macrophages, sesquiterpenes

## Abstract

This study examined whether the antidermatophytic activity of essential oils (EOs) can be used as an indicator for the discovery of active natural products against Leishmania amazonensis. The aerial parts of seven plants were hydrodistilled. Using broth microdilution techniques, the obtained EOs were tested against three strains of dermatophytes (Trichophyton mentagrophytes, Microsporum gypseum and Microsporum canis). To compare the EOs antifungal and antiparasitic effects, the EOs activities against axenic amastigotes of L. amazonensis were concurrently evaluated. For the most promising EOs, their antileishmanial activities against parasites infecting peritoneal macrophages of BALB/c mice were measured. The most interesting antifungal candidates were the EOs from Cymbopogon citratus, Otacanthus azureus and Protium heptaphyllum, whereas O. azureus, Piper hispidum and P. heptaphyllum EOs exhibited the lowest 50% inhibitory concentration (IC_50_) values against axenic amastigotes, thus revealing a certain correspondence between both activities. The P. hispidum EO was identified as the most promising product in the results from the infected macrophages model (IC_50_: 4.7 µg/mL, safety index: 8). The most abundant compounds found in this EO were sesquiterpenes, notably curzerene and furanodiene. Eventually, the evaluation of the antidermatophytic activity of EOs appears to be an efficient method for identifying new potential drugs for the treatment of L. amazonensis.

A promising, current strategy for the discovery of bioactive natural products is based on bioinspiration. The aim is to understand the functional role of secondary metabolites in living organisms and transpose the desirable properties to a corresponding research field. Gaining inspiration from the abilities of plants or microorganisms to produce adapted bioactive molecules under environmental pressure has led to some promising results, for example, in the search for antibiotic or antiviral agents (Pan et al. 2010 ) or natural antifungal products (Basset et al. 2012). Essential oils (EOs) are composed of volatile odoriferous compounds which play a major role in the complex interactions taking place between plants and pollinators, herbivorous insects, larger herbivores or microorganisms. In particular, they are among the most efficient antimicrobial compounds of plants' chemical defense systems (Unsicker et al. 2009). This antimicrobial activity points to the use of a bioinspired strategy for the search for antifungal compounds within EOs. In the context of the growing interest in the uses of medicinal plants and, especially, EOs as new antifungal agents (Rios & Recio 2005), we examined seven EOs obtained from particularly fragrant plant species from French Guiana, presaging a distinctive richness and complexity of volatile compounds that potentially exhibit antimicrobial activity. In addition, the extensive search for new drugs to treat leishmaniasis is definitely necessary because the limited number of currently available products present noticeable side effects and the resistance to these products is increasing (Rocha et al. 2005). Known antifungal drugs such as amphotericin B, miltefosine and azoles have also demonstrated activity against *Leishmania* parasites (Moskowitz & Kurban 1999, Tong et al. 2007, Shakya et al. 2011 b). These successful results led to the development of the "therapeutic switching" or "alternative drug use" strategy (Shakya et al. 2011a). In accord with this perspective, we evaluated the antileishmanial properties of selected antidermatophytic EOs. To our knowledge, the correspondence between these two activities has never been investigated for these particular natural products.

## MATERIALS AND METHODS

*General remarks* - *Plant material and sample preparation* - Seven EOs were obtained: *Achetaria guianensis* Pennell (Scrophulariaceae, leaves and stems), *Cymbopogon citratus* (DC.) Stapf (Poaceae, leaves), *Mikania micrantha* Kunth (Asteraceae, aerial parts), *Otacanthus azureus* (Linden) Ronse (Plantaginaceae, aerial parts), *Piper hispidum* Sw. (Piperaceae, leaves), *Protium heptaphyllum* (Aubl.) Marchand (Burseraceae, fresh green fruits), *Vouacapoua americana* Aubl. (Fabaceae, wood). Herbarium vouchers (respectively Silland 8, 40, 31, 30, 23, 20 and Rodrigues 6) were deposited in the French Guiana herbarium (CAY), where specialists (S Gonzalez, MF Prevost, F Crozier) and members of our laboratory (E Houël, A Rodrigues) confirmed identification. Plants were collected in French Guiana near Regina, Matoury and Cayenne, mainly during the rainy season (April-July) except for *A. guianensis* which was collected during the dry season (November). The fresh parts collected from each plant were hydrodistilled and the EOs were stored at -18ºC until the subsequent analyses were performed. The material under study is endotoxin free.

*Nuclear magnetic resonance (NMR) spectroscopy* - The ^1^H NMR spectra and ^13^C NMR spectra were recorded at 400 MHz and 100.6 MHz, respectively, using a Varian 400 MR spectrometer equipped with a 5 mm inverse probe (Auto X PGF ^1^H/^15^N-^13^C). The EOs were dissolved in deuterated chloroform (CDCl_3_) in 5 mm tubes.

*Gas chromatography-mass spectrometry (GC*-*MS) analysis* - A Varian 450-GC fitted with a MS240 ion-trap MS and a Combipal autosampler was used for the GC-MS analysis. The GC was run with a non-polar Varian FactorFour VF-5ms column (30 m × 0.25 mm ID, 0.25 μm film) commonly used for the analysis of VOCs. The injection volume (EO dissolved in chromatography-grade hexane) was 1 µL. Helium was used as the carrier gas at a constant flow of 1 mL/min. The column temperature increased from 50-150ºC at 4ºC/min, then from 150-175ºC at 1.5ºC/min and from 175-300ºC at 20ºC/min for a total analysis time of 58.42 min. The injector temperature was set to 250ºC and the injection was made with a split ratio of 1/50 during the whole run. The MS was operated in the electron impact mode at 70 eV, with a scan range of 40-400 m/z. The temperatures were set to 200ºC for the ion trap, 50ºC for the manifold and 305ºC for the transfer line. The relative proportions of constituents of the EOs were expressed as the percentages obtained by peak area normalisation.

*Component identification* - The identification of the components of the EOs was based on the following: (i) GC retention indices (RI) on a non-polar column, (ii) computer matching with commercial mass spectral libraries (NIST 98 MS, ADAMS) (Adams 2007 ), (iii) comparisons of RI and spectra with those from previous work (Courtois et al. 2009, Houël et al. 2014) and from an in-house library of analyses of commercial EOs of known composition (Aromazone) and (iv) NMR spectroscopy.

*Fungal strains* - One clinical isolate of a *Trichophyton* species (*Trichophyton mentagrophytes* LMGO 1931) and two clinical isolates of *Microsporum* species (*Microsporum gypseum* LMGO 10 and *Microsporum canis* LMGO 22) were kindly provided by Dr Maria do Rosario Silva (University Hospital, Federal University of Goiás, Brazil). The cultures were maintained on potato dextrose agar and were cultured onto a new agar plate at 28ºC for five days prior to antimicrobial tests.

*Parasites and cultures* - A cloned line of *Leishmania amazonensis* (strain MHOM/BR/76/LTB-012) was used in all of the experiments. An axenically grown amastigote form of *L. amazonensis* was maintained by weekly subculturing in MAA20 medium at 32º+/-1ºC in 25 cm^2^ tissue culture flasks with 5% CO_2_ and supplemented with 20% heat-inactivated foetal bovine serum (FBS), as previously described (Estevez et al. 2007).

*Minimal inhibitory concentration (MIC)* - The standard microdilution test was used to determine the MIC of the EOs. The experimental details were similar to those described previously (Houël et al. 2014). All assays were conducted in triplicate.

*Cytotoxicity assay using VERO cells* - VERO cells (African Green Monkey kidney epithelial cells) were seeded (5 x 10^5^ cells mL^-1^, 100 µL per well) in 96-well flat-bottom plates at 37ºC with 5% CO_2_. RPMI-1640 medium without phenol red and supplemented with 10% heat-inactivated FBS was used. After the EOs were added, the cells were cultured for 48 h. The effects of the treatments were determined using the 3-(4,5-dimethylthiazol-2-yl)-2,5-diphenyltetrazolium bromide (MTT) viability assay. Four hours after the addition of MTT, 100 µL of lysis buffer [50% isopropanol, 10% sodium dodecyl sulfate (SDS)] was added and the cells were shaken for 30 min at room temperature (RT). The optical density (OD) was read at 595 nm using a 96-well plate reader (Chameleon, Hidex; Finland). All experiments were performed in triplicate. The median toxic dose (TD_50_) values were determined using linear regression analysis. The TD_50_ was defined as the concentration of the test sample that resulted in a 50% reduction of absorbance compared to controls.

*Activity on axenic amastigotes* - All experiments were performed in triplicate. The in vitro leishmanicidal activities of the EOs were determined in axenic cultures of the amastigote form of *L. amazonensis*. To estimate the 50% inhibitory concentration (IC_50_) of the extracts, the MTT was used as previously described (Estevez et al. 2007). Results were expressed as the percentage reduction of parasite burden compared to the level in untreated control wells and the IC_50_ was determined from the concentration response curves (Excel software). Briefly, axenically grown amastigotes during the late log phase of growth were seeded in 96-well flat bottom microtitre plates. EOs, dissolved in dimethyl sulfoxide (DMSO), were added at final concentrations ranging from 100-10 µg/mL. The final DMSO concentration was never > 0.1%. After 72 h of incubation, 10 µL of MTT (10^-3^ µg/mL) was added to each well and the plates were further incubated for 4 h. After these 4 h, the enzymatic reaction was stopped with 100 µL of a 50% isopropanol and 10% SDS solution and the plates were incubated for an additional 30 min under agitation at RT. Finally, the OD was read at 595 nm with a 96-well scanner (Bio-Rad). The reference compound was amphotericin B.

*Activity on Leishmania infected macrophages* - Mouse peritoneal macrophages were collected in cold phosphate buffered saline (pH 7.2). One million macrophages collected from BALB/c mouse were allowed to adhere to 12 mm diameter glass coverslips (105 cells per coverslip). Coverslips were transferred into 16 mm diameter well of 24-well plates. Each well contained 0.5 mL of RPMI-10% foetal calf serum (FCS) supplemented with 100 µg/mL streptomycin and 100 UI/mL penicillin. The adherent cells were cultured at 37ºC under 5% CO_2_ for 3 h. Then the plates were washed with RPMI supplemented with hepes, without FCS to eliminate non-adherent cells. The supernatant was replaced by 0.5 mL/well of fresh medium RPMI + 10% FCS + antibiotics before infection by *L. amazonensis* amastigotes at a ratio of five infecting organisms to one host cell. After a 2 h contact, the drugs to be tested were added to the culture and maintained at 37ºC under 5% CO_2_ for 48 h. Then, plates were fixed with methanol and stained with 10% Giemsa's stain (Merck). They were fixed up with Gurr Resin (BDH Chemicals Ltd, England). Macrophages with and without parasites were counted under 40X magnification. For each triplicate assay, the survival index of amastigotes was calculated relative to the control.

*Ethics* - Mice were treated according to French legislation (Ethical Committee, US006 CREFFE, registered CEEA-122).

## RESULTS

*In vitro antifungal activity of EOs* - The in vitro antifungal activities of the seven EOs are presented in Table I. To improve the clarity of the results, a score representing the global antifungal activity was attributed to each EO. A MIC greater than 500 µg/mL received a 0, a MIC of 500 µg/mL received a 1, a MIC of 250 µg/mL received a 2 and each subsequent reduction in MIC by a factor of 2 increased the number of the score by 1. According to these scores, the most active antifungal EOs are those of *C. citratus*, with a score of 17 (representing MICs of 16, 8 and 62 µg/mL against *M. gypseum*, *T. mentagrophytes* and *M. canis*, respectively), *O. azureus* (16) and *P. heptaphyllum* (13). The EOs of *V. americana* (9) and *P. hispidum* (8) also exhibited high antifungal activity with MICs in the 62-500 µg/mL range*. *The EOs of *M. micrantha* (5) and *A. guianensis* (0) exhibited weak to non-existent activity against the selected dermatophytic filamentous fungi (MIC values from 125 to > 500 µg/mL). Among the remarkably active oils, the MICs recorded for the effects of the *C. citratus* and *O. azureus* EOs were as low as 8 µg/mL against *T. mentagrophytes* and 16 µg/mL against *M. gypseum*. These values were the same as that of the reference antifungal agent fluconazole against *T. mentagrophytes* and only twice that obtained for fluconazole against *M. gypseum*; both values were 8 µg/mL for fluconazole.

**TABLE I:** Minimum inhibitory concentrations (μg/mL), antileishmanial activity against axenic amastigotes [50% inhibitory concentration (IC50) (μg/mL)] and cytotoxicity [median toxic dose (TD50) (μg/mL), BALB/c mice peritoneal macrophages and VERO cells measured for the selected essential oils (EOs) and the reference antifungal (itraconazole and fluconazole) and antileishmanial (amphotericin B) drugs

	Dermatophytic filamentous fungi					*Leishmania amazonensis*		Cytotoxicity		
EO	*Microsporum gypseum* LMGO 10	*Trichophyton mentagrophytes* LMGO 1931	*Microsporum canis * LMGO 22	Antifungal activity (score)		IC_50_ axenic amastigotes		TD_50_ BALB/c mice peritoneal macrophages	TD_50 _ VERO cells	SI^*a*^
*Achetaria guianensis*	> 500	> 500	> 500	0		6.3		32.5	30.7	5
*Cymbopogon citratus*	16	8	62	17		5.3		25.2	10.7	5
*Mikania micrantha*	250	125	> 500	5		6.8		50.8	93.2	7
*Otacanthus azureus*	16	8	125	16		0.7		35.5	> 100	51
*Piper hispidum*	125	62	500	8		3.4		35.5	> 100	11
*Protium heptaphyllum*	62	31	62	13		3.7		71.2	> 100	19
*Vouacapoua americana*	62	62	500	9		7.2		34.3	34.5	5
Itraconazole	0.5	0.5	4	-		NT		NT	> 10	NT
Fluconazole	8	8	NT	-		NT		NT	283.2	NT
Amphotericin B	NT	NT	NT	-		0.3		3.7	1	12

a: antileishmanial selectivity index (SI) defined as SI = TD50 (BALB/c mice peritoneal macrophages)/IC50 (L. amazonensis axenic amastigotes); NT: not tested.

*Effects of EOs activities on the growth of axenic amastigotes and cytotoxic effects on BALB/c mice peritoneal macrophages* - The seven EOs were concurrently tested against axenic amastigotes of *L. amazonensis*. The results are presented in Table I. While the EOs from *C. citratus*, *O. azureus* and *P. heptaphyllum* were the most interesting antifungal candidates, the EOs of *O. azureus*, *P. hispidum* and *P. heptaphyllum* exhibited the lowest IC_50_ against axenic amastigotes, thus revealing a certain level of correspondence between both activities. A very high in vitro activity (IC_50_ of 0.7 µg/mL) was measured for the *O. azureus* EO. This value is in the same range as the one obtained for the reference compound amphotericin B (0.3 µg/mL). The *P. heptaphyllum* and *P. hispidum *EOs were also remarkably active against the parasite (IC_50_ values of 3.7 and 3.4 µg/mL, respectively). Overall, IC_50_ values < 10 µg/mL were recorded for all seven EOs.

We also evaluated the selectivity index (SI) based on the toxicity measured on healthy macrophages. The most interesting oil in this respect was *O. azureus*, which had an SI value of 51. Among the other oils identified as the most active against *L. amazonensis*, the EOs of *P. heptaphyllum* and *P. hispidum* exhibited reasonably high selectivity indices of 19 and 11, respectively, which were comparable to the value of 12 obtained for amphotericin B. In contrast, even though the *C. citratus* EO was identified as the most potent antidermatophytic product and also exhibited high antileishmanial activity, this EO was shown to have a low SI of only 5 and thus is not as good of a candidate as the other three EOs with relatively high SIs.

Based on these results, the EOs of *O. azureus*, *P. heptaphyllum* and *P. hispidum* were selected to be further evaluated for their antileishmanial activity against parasites infecting BALB/c mice peritoneal macrophages.

*Cytotoxicity assay on VERO cells* - The toxicities of the EOs towards VERO cells are presented in Table I. Interestingly, the three most antileishmanial EOs (*O. azureus*, *P. heptaphyllum* and *P. hispidum*) exhibited no cytotoxicity against VERO cells (TD_50_ > 100 µg/mL). The *M. micrantha* EO was also not cytotoxic. However, the EOs of *C. citratus*, *A. guianensis* and *V. americana* were all cytotoxic towards VERO cells at concentrations between 10-35 µg/mL. These results confirmed the selection of *O. azureus*, *P. heptaphyllum* and *P. hispidum* for further evaluation.

*Leishmanicidal activity in L. amazonensis-infected BALB/c mice peritoneal macrophages* - To evaluate the potential of the three selected EOs as clinical antileishmanial agents, they were added to a culture media containing *L. amazonensis*-infected BALB/c mice peritoneal macrophages (Table II). Notably, the *P. hispidum* EO exerted the highest leishmanicidal effect, with an IC_50_ of 4.7 µg/mL. While this value is superior to the one recorded for amphotericin B (0.6 µg/mL), the safety indices are very similar.

**TABLE II:** Antileishmanial activity [50% inhibitory concentration (IC50) (μg/mL)] against infected BALB/c mice peritoneal macrophages, safety index for BALB/c mice peritoneal macrophages and infection reduction index at the maximum concentration measured for the three most promising essential oils (EOs) and the reference antileishmanial drug (amphotericin B)

	*Leishmania amazonensis*		
EO	IC_50_ BALB/c mice infected peritoneal macrophages	Safety index on macrophages	Infection reduction index (%) (maximum concentration, µg/mL)
*Otacanthus azureus*	16.1	2	64.7 (20)
*Piper hispidum*	4.7	8	97.5 (20)
*Protium heptaphyllum*	34.9	2	59.6 (40)
Amphotericin B	0.6	6	(2)

The infection reduction indices were also calculated. In this respect, the *P. hispidum* EO was the most active causing a 97.5% reduction of the infection at a dose of 20 µg/mL. The same activity was obtained at 2 µg/mL for amphotericin B.

*Determination of the composition of the P. hispidum EO by GC-MS and NMR analyses* - As the *P. hispidum* EO was identified as the most promising product in the infected macrophages model it was submitted to detailed chemical analysis. There were 64 compounds identified in the *P. hispidum* EO, accounting for 90.5% of the composition of the oil. The details of the identifications and relative concentrations of the compounds found in the hydrodistilled oil of *P. hispidum* are reported in Supplementary Table. The compounds representing more than 1% of the EO are described in Table III. The chemical composition of the *P. hispidum* EO obtained in this study revealed that sesquiterpenes are the most abundant compounds; the five most abundant compounds identified by the GC/MS analysis were curzerene (15.7%), germacrene B (10.9%), α and β-selinene (10.5 and 7.6%, respectively) and β-caryophyllene (4.7%) (Figure). It is known that curzerene can be produced from furanodiene through a thermal Cope rearrangement, with 1,4-dienes being involved in this [3.3]-sigmatropic reaction due to the high temperatures that occur during the injection of the sample into the GS (Baldovini et al. 2001). The comparison of the ^13^C NMR spectra of the crude oil with the data in the literature allowed us to confirm the presence of curzerene, but also revealed the presence of the heat-sensitive compound furanodiene in the crude EO, even if the relative proportions could not be evaluated (Baldovini et al. 2001). Hence, the curzerene identified in the GC/MS analysis in fact originates from curzerene already present in the EO and from its precursor, furanodiene; thus, the quantitative data are affected by the contribution from the Cope rearrangement.

**TABLE III:** Main components (> 1 %) of the Piper hispidum essential oil identified by the gas chromatography-mass spectrometry analysis

RI^*a*^	Composition (%)	Compound	Courtois et al. (2009)	RI Adams (2007)	Houël et al. (2014)
935	1	α-pinene	940	932	936
980	1.4	β-pinene	985	974	981
1379	1.2	α-copaene	1381	1374	1380
1391	2.6	β-elemene	1385	1389	1391
1423	4.7	β-caryophyllene	1427	1417	1424
1432	1.5	γ-elemene	1432	1434	-
1434	1.2	β-copaene	-	1430	-
1460	2.2	α-humulene	1462	1452	1461
1476	1.1	selina-4,11-diene	1482	-	-
1493	7.7	β-selinene	1496	1489	-
1497	15.7	curzerene^*b*^	-	1499	-
1499	10.5	α-selinene	1496	1498	1499
1515	1.1	γ-cadinene	1518	1513	-
1519	3.4	δ-cadinene	1521	1522	-
1524	1.4	calamenene (UI)	-	1521/1528	1524
1561	10.9	germacrene B	1567	1559	-
1597	1.4	viridiflorol	-	1592	1599
1620	1.3	1,10-di-*epi*-cubenol	-	1613^*c*^	-
1657	3.9	7-*epi*-a-eudesmol	-	1662	-
1660	4.6	junicedranone	-	1664	-
Total	78.9	-	-	-	-

a: the identified constituents are listed in their order of elution from a non-polar column (Varian FactorFour VF-5ms); b: from curzerene and furanodiene. Quantitative data are affected by thermal rearrangement; c: Cicció and Chaverri (2008); RI: retention indices; UI: undetermined isomer.

**Figure:**
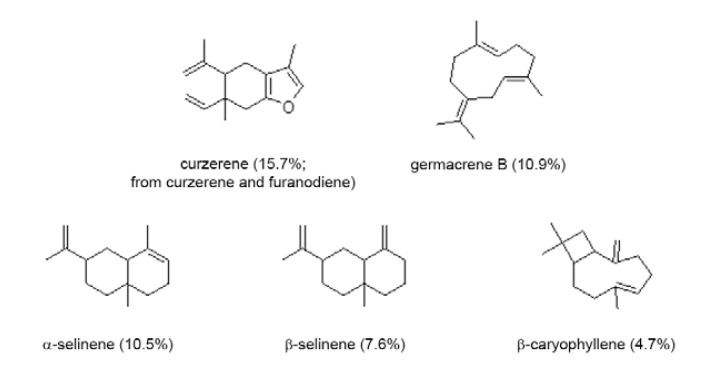
Main components of the Piper hispidum essential oil identified by gas chromatography-mass spectrometry.

## DISCUSSION

The three most active antifungal EOs were those from *C. citratus*, *O. azureus* and *P. heptaphyllum*. The EO of *C. citratus* has largely been described as antifungal (Shin & Lim 2004 , da Silva et al. 2008 ). In our study, the *C. citratus* EO was mainly composed of neral (31%) and geranial (56%), corroborating the already well-known antifungal activity of citral, known to act by forming a charge transfer complex with an electron donor of fungal cells and thus causing fungal death (da Silva et al. 2008). The antidermatophytic activity and chemical composition of the *O. azureus* EO has been further studied elsewhere (Houël et al. 2014). It was shown to be largely composed of sesquiterpenes, with the main component being β-copaen-4-α-ol (23%), alongside α-humulene (10.6%), α-copaene (8.8%), myrtenal (5.6%), viridiflorol (5.1%) and trans-pinocarveol (4.3%). Concerning the EO of *P. heptaphyllum*, we have demonstrated for the first time that the oil extracted from fresh green fruits is a highly potent antifungal agent against dermatophytic filamentous fungi. Moreover, this oil exhibited no cytotoxicity against VERO cells (TD_50_ > 100 µg/mL). Further studies should be conducted on this EO to confirm the fact that it represents a promising product for the treatment of human superficial dermatomycoses. In our extract, the *P. heptaphyllum* EO was mainly composed of limonene (82%), along with small proportions of other monoterpenes (α-pinene 5.4%, β-pinene 2.5%, *p*-cymene 1.5%, *trans*-carveol 0.9%, β-myrcene 0.7% and carvone 0.7%). This composition differs from the one already published for immature fruits (Pontes et al. 2007), which indicated that the primary component was α-terpinene. We tested the three main compounds for their antidermatophytic activities, but all of them were inactive. Similarly to the *O. azureus* EO, the antifungal activity could thus be due to a synergistic effect of multiple compounds, as that described for limonene and α-pinene on *S. cerevisiae* or to the activity of a minor component (Tserennadmid et al. 2011). In addition, the EOs of *V. americana* and *P. hispidum* also exhibited significant antifungal activity*. *Antidermatophytic as well as antimicrobial activity have already been described in the literature for some *P. hispidum* EOs (Morales et al. 2013, Tangarife-Castaño et al. 2014).

The seven oils were concurrently tested against axenic amastigotes of *L. amazonensis*. Infections with this parasite result in a clinical spectrum of manifestations that includes all three forms of leishmaniasis (cutaneous, mucosal and visceral) (Rocha et al. 2005). Of the three most antifungal EOs, two of them (*O. azureus* and *P. heptaphyllum*) also exhibited remarkable antileish- manial activities, especially *O. azureus* (IC_50_ 0.7 µg/mL). Concerning *O. azureus* EO, none of its main components has to our knowledge been clearly identified as antileishmanial. However, *P. heptaphyllum* EO was shown to be mainly composed of limonene, recently demonstrated to attack the plasma membrane of the parasite (Camargos et al. 2014). A third oil, that of *P. hispidum* leaves, was also identified as a potent antiamastigote agent with an IC_50_ of 3.4 µg/mL. We had previously observed that this EO exhibited significant antifungal activity, demonstrated by a high score for activity (8) and MIC values ranging from 62-500 µg/mL. Notably, the *C. citratus* EO was identified as the most potent antidermatophytic product and also demonstrated significant anti-amastigote activity. This dual activity against both filamentous dermatophytic fungi and *Leishmania* sp. amastigotes has already been observed with miltefosine, amphotericin B and azoles (Moskowitz & Kurban 1999, Tong et al. 2007, Shakya et al. 2011b). In fact, amphotericin B and azoles, which were initially developed as antifungals and are now used (or have been successfully tested) against *Leishmania *sp.*,* are both involved in interactions with the sterols of fungal membranes that lead to cell death. The former cause death by inhibiting the demethylation of lanosterol and the latter disrupts the synthesis of ergosterol (Ghannoum & Rice 1999). The antileishmanial activities of these molecules is thus due to the relatively high content of ergosterol in the membranes of* Leishmania *and the result of similar mechanisms to those occurring in fungi (Gebre-Hiwot & Frommel 1993). In addition, miltefosine interferes with phospholipid metabolism (Tong et al. 2007). Targeting antifungal natural products potentially having an effect on *Leishmania* cell membrane is thus relevant (Bou et al. 2014). To our knowledge, this is the first time that such a correspondence in activity has been shown for EOs, even though the modes of actions should be investigated further.

At this stage of the study, the EOs found to exhibit both the best antifungal activity and the lowest IC_50_ against axenic amastigotes were those of *O. azureus*, *C. citratus*, *P. heptaphyllum* and *P. hispidum*. The toxicities of these EOs towards BALB/c mice peritoneal macrophages were then also evaluated. The best selectivity indices regarding antiparasitic activity were obtained for the *O. azureus* (71), *P. heptaphyllum* (19) and *P. hispidum* (11) EOs. These three EOs were also non-toxic to VERO cells, whereas the *C. citratus* EO had a TD_50_ of 30.7 µg/mL. It should be noted that the *O. azureus* and *P. heptaphyllum* EOs or extracts have never been described as antileishmanial agents. Though *P. hispidum* extracts are already known for their antileishmanial activity against *L. amazonensis* (Estevez et al. 2007, Ruiz et al. 2011), this is the first time that these properties are described for the EO.

To confirm the potential use of these EOs as antileishmanial agents and corroborate the results indicating that the examination of alternative uses of natural antifungal products could lead to the discovery of promising anti- leishmanial drugs, we evaluated the activity of these last three oils on *L. amazonensis*-infected BALB/c mice peritoneal macrophages, excluding the *C. citratus* EO because of its relative toxicity. As *Leishmania* parasites survive and multiply within mammalian macrophages, this model produces results more closely related to in vivo results and a therapeutic drug can only demonstrate activity if it can cross the host cell membrane and act on the intracellular amastigotes (Kyriazis et al. 2013, Rodrigues et al. 2013). The EO of *P. hispidum* was clearly the most potent and promising oil, with an IC_50_ of 4.7 µg/mL and a safety index of 8, a value superior to the one calculated for the reference drug amphotericin B. This EO reduced the infection by 97.5% at 20 µg/mL. The present results confirm the interest of natural compounds study, including crude extracts or fractions, for the discovery of potent antileish- manial compounds, as underlined by Rabito et al. (2014).

The compositions of some *P. hispidum* EOs have already been described in the literature (Pino et al. 2004, Benitez et al. 2009, Cruz et al. 2012 , Assis et al. 2013, Morales et al. 2013). Our findings are consistent with previous results; the *P. hispidum* EO extracted in this study was mainly composed of sesquiterpenes, though the proportions of oxygenated sesquiterpenes and sesquiterpene hydrocarbons are highly variable. This result possibly being due to seasonal or environmental variations (Figueiredo et al. 2008, Duarte et al. 2009), repeating this study on new *P. hispidum* collections and extractions could therefore help to assure the correlation between the EO composition and biological activity. Curzerene has been previously identified in some oils, but never as the major component (Benitez et al. 2009). In our hands, the *P. hispidum* EO was shown to contain both curzerene and its precursor furanodiene and the relative proportion of curzerene calculated by GC analysis was thus overestimated. Other furanosesquiterpenes were detected by GC/MS and NMR analysis but could not be identified. Curzerene has already been found in other antileishmanial EOs (Rodrigues et al. 2013) and, considering our findings, furanosesquiterpenes could contribute to the antileishmanial activity of the *P. hispidum* EO. Moreover, β-caryophyllene, which accounts for 4.7% of this EO, is known to be an antileishmanial compound, possibly having an antileishmanial activity associated with the inhibition of the biosynthesis of cellular isoprenoids (Santos et al. 2008). According to these data and those concerning the other active EOs, correlating the chemical composition of the EOs and their biological activity, for example through a metabolomic approach, could lead to valuable information. Indeed, β-caryophyllene representing in particular only 0.52% of *O. azureus* EO (Houël et al. 2014) and not having been identified in *P. heptaphyllum* EO, other potent antileishmanial molecules could thus be revealed.

In conclusion, the bioinspired selection of fragrant species successfully led to the identification of strongly antifungal compositions. This study also demonstrated the significant antileishmanial potential of the EO of *P. hispidum* against *L. amazonensis*, pending confirmation with in vivo assays. It would also be an interesting perspective to perform synergy studies between the most abundant compounds and antileishmanial chemotherapeutics as amphotericin B, as well as further investigate the role of synergy concerning biological activity and selectivity of the crude oil itself. Eventually, the evaluation of the antidermatophytic activities of EOs appears to be a promising strategy for the discovery of new natural antileishmanial products, a significant achievement within the context of the alternative drug use, especially considering factors such as the low cost, high accessibility, high availability and reduced cytotoxicity of these products.
